# A Case-Based Review of the Management of Penetrating Brain Trauma

**DOI:** 10.7759/cureus.1342

**Published:** 2017-06-12

**Authors:** Jason Milton, Alex Rugino, Kailash Narayan, Chris Karas, Victor Awuor

**Affiliations:** 1 Neurosurgery, Ohio Health

**Keywords:** trauma, traumatic brain injury, penetrating trauma

## Abstract

Principles of penetrating head trauma management were established by Harvey Cushing in relation to the management of penetrating brain injuries of World War One. Cushing radically debrided the scalp and skull and aggressively irrigated wound tracks to remove foreign bodies. He would then obtain water-tight closure. Cushing significantly decreased infection rates which reportedly limited the major cause of mortality due to penetrating head injuries. Many advances have been made by contributions from World War Two, Korean War, Vietnam War, and Iran/Iraq conflicts. Early radical decompression, with conservative debridement and duraplasty applied to blast-induced penetrating injuries during Operation Iraqi Freedom, has resulted in increased survivability and neurological improvement. Each advance in the management of these injuries is based upon more effectively addressing one or more components of Matson’s tenets.

This case series reviews the successful management of three patients that presented to a level I trauma center with a penetrating head injury from high-velocity projectiles. Management principles of each patient begin with a proper patient assessment, application of Matson’s tenets from the time of injury, and airway control. Surgical management is based upon adherence to Grahm’s Guidelines which emphasize criteria centered upon post-resuscitative Glasgow Coma Scale score and appropriate imaging.

This case series suggests that proper patient evaluation, adherence to Matson’s tenets and to Grahm’s Guidelines, and appropriate patient selection for operative management leads to improved survival of patients with penetrating head trauma from high-velocity projectiles.

## Introduction

Gunshot wounds (GSWs) account for the majority and have the highest mortality of all penetrating brain injuries (PBI). Approximately two-thirds will die at the scene, and up to half of the survivors will then die in the first 24 hours. Overall, 90% of civilian GSWs are lethal [1]. Principles to guide treatment of GSWs are outlined by Grahm (Table 1) and Matson (Table 2) [2-3].

**Table 1:** Grahm's principles.

Glasgow Coma Score	Surgery vs nonoperative management
3-5	Not associated with satisfactory outcome
5-7	Should be managed nonoperatively if their injury is multilobar, transventricular, or in the dominant hemisphere
>7	Should be considered for operative management

**Table 2:** Matson's tenets.

Tenet	Current application
I. Save life	Advanced trauma life support/advanced cardiac life support/far forward homeostasis and hemicraniectomy
II. Prevent infection	Watertight dural closure
III. Preserve nervous system function	Prevention of secondary neurologic injury through advanced neurocritical and neurointerventional care
IV. Restore anatomic function	Restore anatomic protection and contour

Initial assessment and management of PBI begin with the typical advanced trauma life support (ATLS) and advanced cardiac life support (ACLS) approach with documentation of entry (and exit) sites, missile fragment identification, Glasgow Coma Scale (GCS), and pupillary reactivity. Prompt neuroimaging is of utmost importance to determine the need for surgical intervention as well as for crude evaluation of possible impacts on the intracranial pressure that may affect monitoring [4]. Beginning anticonvulsant therapy is controversial, but may be started for concerning lesions and is recommended to be continued for seven days [4]. Antibiotics are typically continued for 10-14 days in patients with retained foreign bodies or with significant risks of infection [4]. As with any penetrating injury in the body, tetanus prophylaxis should be administered as soon as able [1]. Surgical management of PBI should ideally be performed within 12 hours of the injury unless there is an obvious mass lesion or active bleeding creating a neurosurgical emergency [4]. The goals of surgery are debridement of devitalized tissue (minimize debridement volume), removal of accessible mass lesions, removal of bone and bullet fragments, hemostasis, watertight dural closure, and adequate scalp closure [1].

## Case presentation

### Case 1

**Figure 1:**
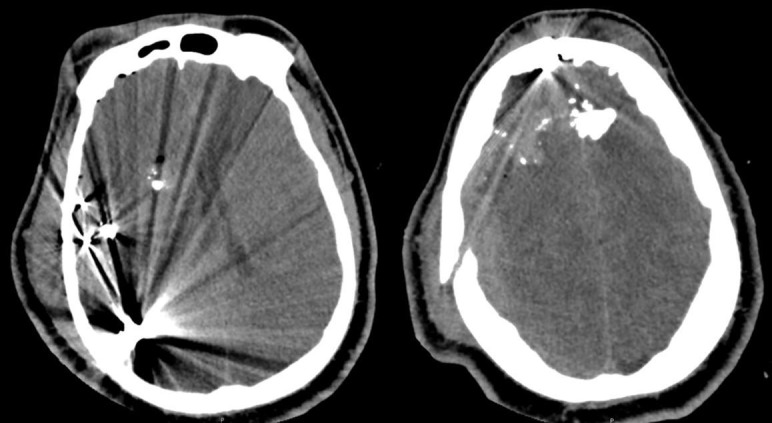
Presenting computed tomography (CT) head which revealed severe intracranial injury from a high-velocity projectile with a trajectory from the right parietal lobe through the left frontal lobe with contusions, ballistic and skull fragments along the pathway. Additional findings included a 7 mm right sided subdural hematoma and a right-to-left midline shift of 5.5 mm.

A 27-year-old male presented with a PBI from a GSW. Initial examination revealed GCS 3T, confounded by paralytics given for intubation. Prior to intubation, he was reported to be moving his right side purposefully. Head computed tomography (CT) revealed severe intracranial injury from a high-velocity projectile with a trajectory from the right parietal lobe through the left frontal lobe with contusions, ballistic, and skull fragments along the pathway. Additional findings included a 7 mm right-sided subdural hematoma and a right-to-left midline shift of 5.5 mm (Figure 1).

The decision was made for immediate craniectomy for decompression. Once the dura was opened, the subdural hematoma (SDH) was evacuated followed by removal of a superficial bone fragment and a ballistic fragment. Along the midline, a bone fracture was repaired, and water-tight dural closure was obtained.

**Figure 2:**
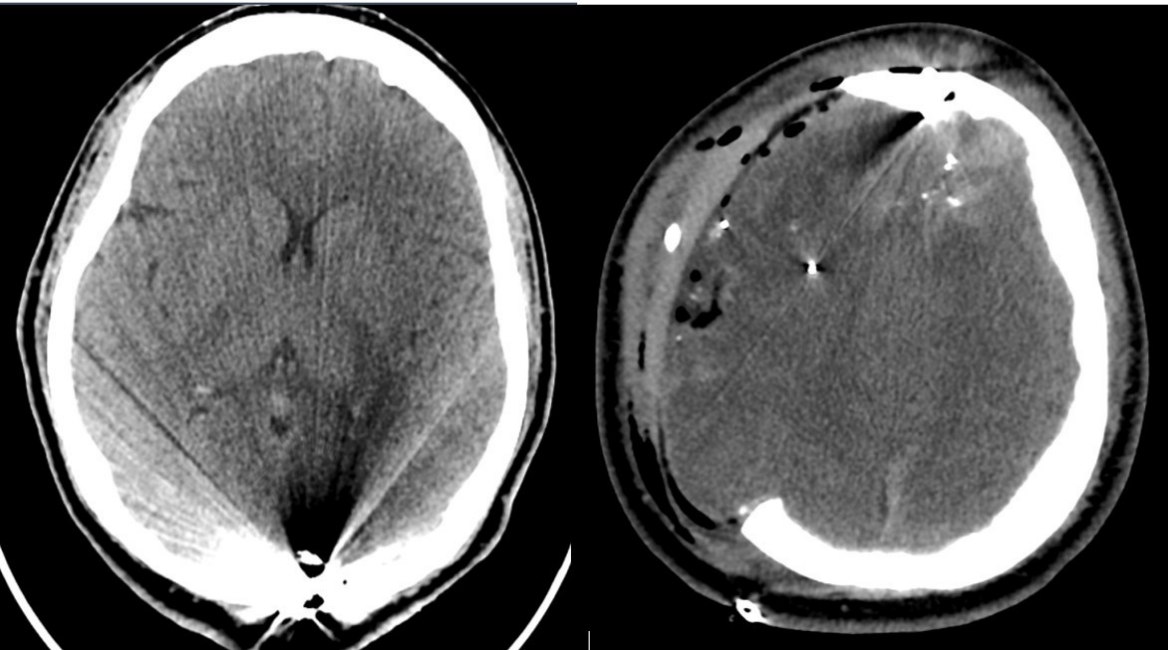
Post-operative computed tomography (CT) head revealed residual subdural hematoma, intraparenchymal hematoma, pneumocephalus, right greater than left edema, craniectomy defect herniation, and a small subgaleal hematoma.

**Figure 3:**
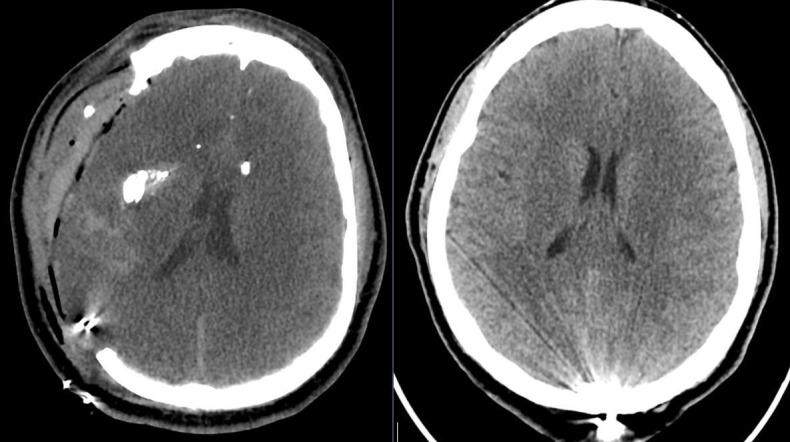
Computed tomography (CT) head revealing improved subfalcine herniation.

Post-operative head CT revealed residual subdural hematoma, intraparenchymal hematoma, pneumocephalus, right greater than left edema, a craniectomy defect herniation, and a small subgaleal hematoma (Figure 2). Some subfalcine herniation was appreciated, but it was noted to be improved from the initial presentation (Figure 3).

The patient was extubated on post-operative day 2. His strength was noted to gradually improve throughout his hospitalization. He was given antibiotics for 24 hours post-operatively and discharged to rehabilitation on post-operative day 6. At his two-week follow-up visit, he was neurologically intact and without acute complaint.

### Case 2

**Figure 4:**
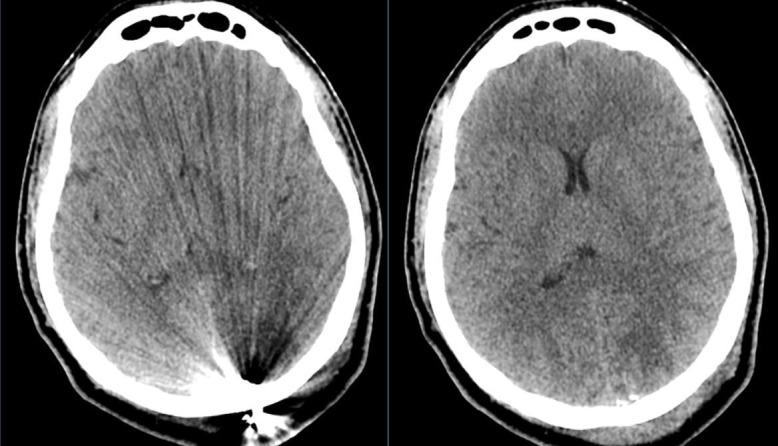
Computed tomography (CT) head revealed a comminuted, depressed left skull fracture, a retained high-velocity projectile in the midline occipital region, and minimal pneumocephalus.

A 32-year-old male presented with a PBI from a GSW to the back of the head without loss of consciousness. Initial examination revealed a punctate hemostatic wound of the occiput, GCS of 15, 3 mm pupils that were reactive to light, and a non-focal neurologic examination. Head CT revealed a comminuted, depressed left skull fracture, a retained high-velocity projectile in the midline occipital region, and minimal pneumocephalus (Figure 4). CT angiography revealed no vascular injury.

Non-operative management included serial neurological checks, avoidance of antiplatelet and anticoagulant medications, elevation of the head of the bed, and close observation. Risks of surgery were determined to outweigh benefit given the proximity to vital vascular structures.

Twenty-four-hour follow-up head CT was stable with a residual small hypodensity in the left occipito-parietal region and extension of a subgaleal hematoma. At discharge on hospital day 3, he was neurologically intact, but was then lost to follow-up. 

### Case 3

A 44-year-old male sustained a PBI from a self-inflicted GSW. In the field, ventricular fibrillation reverted to sinus rhythm after 3 minutes of advanced life care. Paralytics were administered and intubation was performed with a resultant GCS of 3T one hour prior to presentation to the trauma bay. Upon presentation, GCS was 11T and 4 mm pupils were reactive to light.

**Figure 5:**
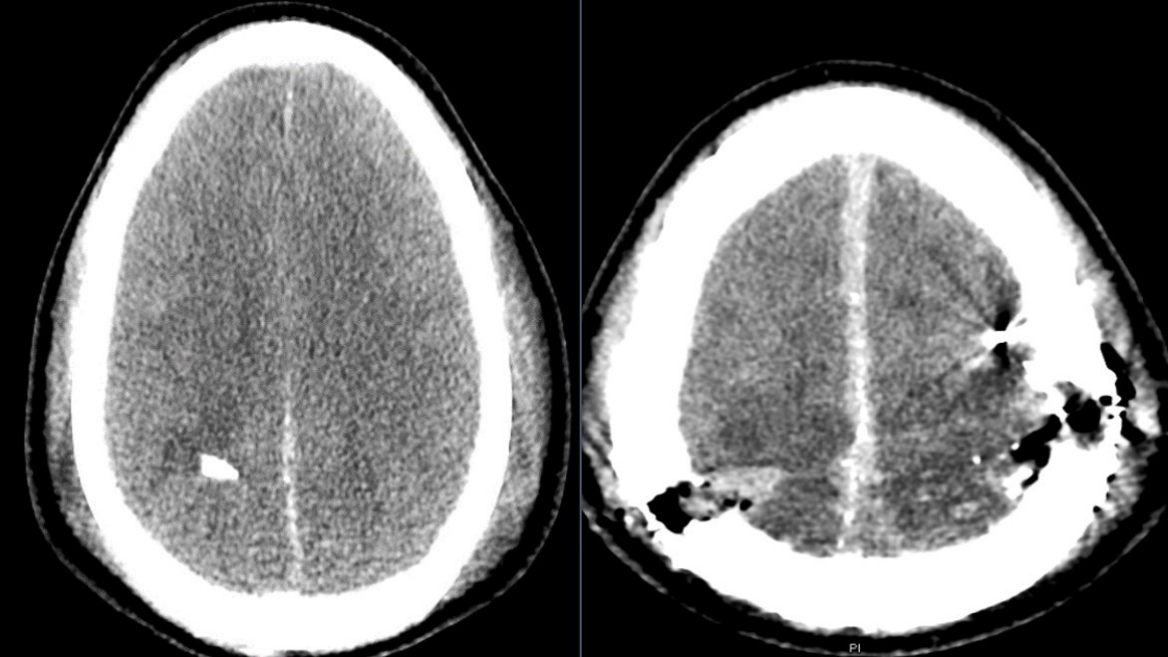
Computed tomography (CT) head revealed multifocal hemorrhagic contusions and edema along the cerebral convexities, complex and nondisplaced skull fractures along the projectile trajectory from the right to left parietal skull. The retained high-velocity projectile was in the left parietal bone.

Head CT revealed multifocal hemorrhagic contusions and edema along the cerebral convexities, complex and nondisplaced skull fractures along the projectile trajectory from the right to left parietal skull. The retained high-velocity projectile was in the left parietal bone (Figure 5).

The decision was made to perform an immediate craniotomy. Initially tissue was debrided from the right parietal region. A left craniotomy then revealed a depressed skull fracture, which was elevated followed by removal of the high-velocity projectile from the left parietal bone with debridement of devitalized brain. Cranioplasty was then performed bilaterally.

**Figure 6:**
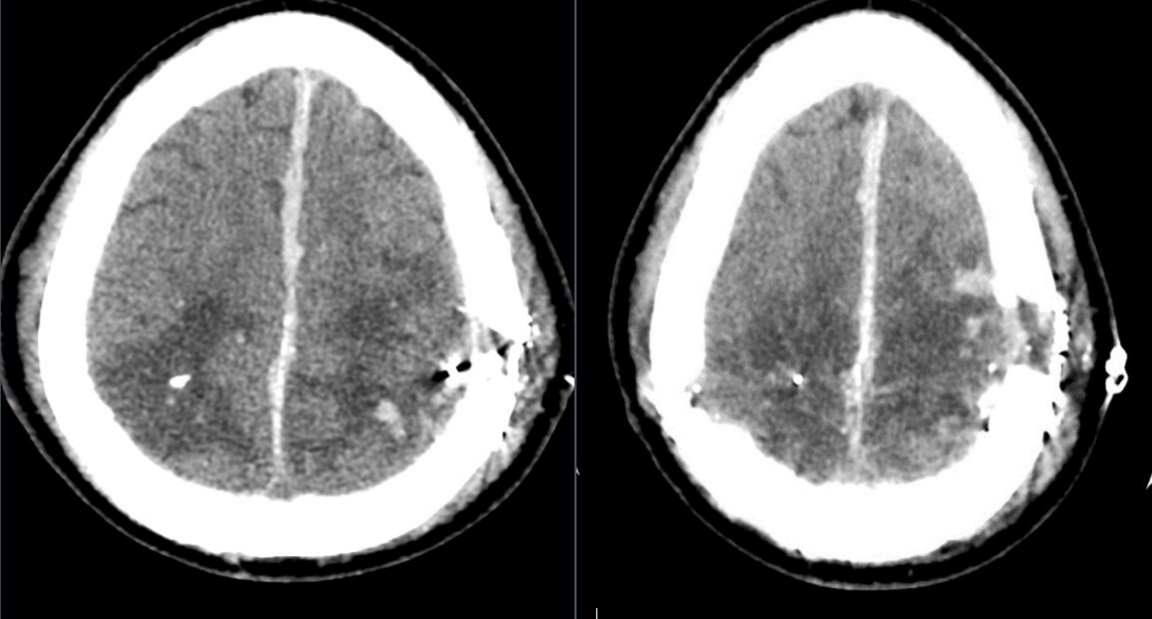
Post-operative computed tomography (CT) head revealed retained projectile and bone fragments along the trajectory. In addition, projectile fragments remained adjacent to the superior sagittal sinus which required anticoagulation due to risk of sinus thrombosis.

Post-operative head CT revealed a retained projectile and bone fragments along the trajectory. In addition, projectile fragments remained adjacent to the superior sagittal sinus which required anticoagulation due to the risk of sinus thrombosis (Figure 6). At discharge to rehabilitation, his physical examination found him to be alert with intact cranial nerve examination, but with quadriparesis.

## Discussion

Three cases of PBI due to civilian GSW were presented as examples of when immediate craniectomy is indicated with resultant outcomes based on management principles outlined by Grahm and Matson.

The patient that did not require resuscitation with an initial examination consistent with unilateral focal neuromotor injury, GCS greater than 5, required extensive surgery unilaterally. He had an excellent outcome with no apparent permanent severe neurologic injury two weeks after discharge. The patient that required resuscitation with an initial GCS of 3T increased to 11T after subsidence of chemical paralysis had severe bilateral injury and underwent extensive surgery bilaterally had a poorer outcome with diffuse severe neuromotor deficits obvious at the time of discharge. The remaining patient was evaluated for surgical intervention and managed non-operatively due to proper assessment of risks and benefits of surgical intervention.

Grahm’s principles and Matson’s tenets were appropriately applied to each of these three cases in which two patients underwent surgical intervention. Although, one must note that cases such as the non-operative patient may create circumstances where the provider must give priority to the separate tenets in the setting of a neurologically intact patient with a risk of infection from a retained projectile.

## Conclusions

Applications of Grahm’s principles and Matson’s tenets are appropriate guidelines for treatment. This case series reviews the outcomes of three appropriately managed patients and highlights a case in which the priority of Matson’s tenets must be established.
